# Ultrathin Co_9_S_8_ nanosheets vertically aligned on N,S/rGO for low voltage electrolytic water in alkaline media

**DOI:** 10.1038/s41598-018-35831-4

**Published:** 2019-02-13

**Authors:** Huan Liu, Cheng-Yan Xu, Yue Du, Fei-Xiang Ma, Yue Li, Jing Yu, Liang Zhen

**Affiliations:** 1 MIIT Key Laboratory of Advanced Structural-Functional Integration Materials & Green Manufacturing Technology, School of Materials Science and Engineering, Harbin Institute of Technology, Harbin, 150001 China; 2 MOE Key Laboratory of Micro-Systems and Micro-Structures Manufacturing, Harbin Institute of Technology, Harbin, 150080 China; 3 School of Materials Science and Engineering, Harbin Institute of Technology (Shenzhen), Shenzhen, 518055 China

## Abstract

Development of hydrogen as clean and efficient energy carrier for future is imperative. Water electrolysis, is considered as one of the most promising ways to realize large-scaled hydrogen production. However, a big obstacle of it is to reduce the electric energy consumption for water oxidation in the anode. Engineering of hierarchical architectures on the electrocatalysts could provide abundant active sites and thus boost the sluggish reaction kinetics of water oxidation. Herein, a sequential synthesis method is developed for *in-situ* growth of ultrathin Co_9_S_8_ nanosheets vertically aligned on N and S co-doped reduced graphene oxide (Co_9_S_8_/N,S-rGO) as novel and efficient electrocatalysts for water splitting. This architecture with vertically aligned ultrathin Co_9_S_8_ nanosheets on N,S/rGO is adopted to facilitate the electron transport and exposure of active sites. Benefiting from the synergetic catalysis between Co_9_S_8_ nanosheets and N,S/rGO, Co_9_S_8_/N,S-rGO presents remarkable electrocatalytic activity towards oxygen evolution with a low overpotential (266 mV to achieve current density of 10 mA cm^−2^), small Tafel slope of 75.5 mV dec^−1^, and good durability in alkaline medium. This remarkable OER electrocatalytic activity is outperforms most of the known noble-metal-free electrocatalysts.

## Introduction

With the increasingly serious consumption of fossil fuels and environmental pollution issue, the searching for clean and sustainable energy sources is imperative^1,2^. Hydrogen as a clean renewable energy is a potential alternative to traditional fossil fuels^3,4^. Electrochemical splitting water into hydrogen (2H_2_O → 2H_2_ + O_2_) is a promising pathway for sustainable hydrogen energy production, where the oxygen evolution reaction (OER) is the bottleneck owing to the sluggish four electron transfer process (4OH^−^ → O_2_ + 2H_2_O + 4e^−^), which is accompanied with the breakage of O-H bond and the combination of O-O bond^5,6^. Great efforts had been expended to exploring high efficiency and stable oxygen evolution electrocatalysts^7,8^. Currently, the most active OER electrocatalysts are oxides of noble metals (e.g., IrO_2_ and RuO_2_), while the concerns related to its high cost and scarcity are big obstacles in the real application^9–11^.

Hence, cobalt based compounds have been exploited as candidate electrocatalysts owing to their abundance in nature, unique d-orbital electronic structure and low Gibbs adsorption energy. These compounds have shown high catalytic activity close to RuO_2_ towards water splitting^12,13^. Several families of cobalt based electrocatalysts, especially cobalt oxides, nitrides, hydroxides, sulfides and phosphides have shown attractive electrocatalytic activity towards water splitting^14–19^. Additionally, a lot of researches about identifying the high-efficient electrocatalytic mechanism of cobalt based electrocatalysts has been carried out through experiments, operando techniques and theoretical calculations^20–22^. Among them, cobalt sulfides, inspired by hydrogenase, have been suggested as promising electrocatalysts for overall water splitting^23,24^. Although the high efficient electrocatalytic activity of cobalt based electrocatalysts has been demonstrated, it is still far from satisfactory comparing with commercial electrocatalysts (e.g., RuO_2_, IrO_2_). The intrinsic low electron transport efficiency and limited number of catalytic active sites of cobalt based electrocatalysts are the main obstacles for further improving the electrocatalytic performance of water splitting^25–27^. Hence, extensive efforts have been dedicated to improve OER electrocatalytic activity of cobalt sulfides. The common methods include morphology control, phase engineering, doping and hybridization. For example, investigators constructed two-dimensional nanosheets, core-shell nanostructure, nickel-iron disulfide and oxhydroxide heterostructure, cubic cobalt sulfide with layered molybdenum disulfide hybridization towards enhancing the electrocatalytic of water splitting^10,28–31^.

It has been demonstrated that reduced graphene oxide (rGO) was an ideal matrix with uniformly grown cobalt sulfides to accelerate electron transmission and buffer volume changes during oxygen evolution process^32,33^. *In-situ* growth nanosheets on rGO had superiority of inhibiting the aggregation of nanosheets and fully exposing surface area to short the electron transport distance. At the same time, this proposal solves the problem that cobalt sulfides as electrocatalysts suffer from dissolution, losing and agglomeration after electrochemical reaction process^34,35^. For example, Li *et al*. reported the *in-situ* growth of transition metal dichalcogenides (TMDs) nanocages encapsulated by rGO, which greatly improved the ion/electron transport along the interfaces and efficiently mitigated volume dilation during LIBs delithiation^34^. In addition, the heteroatoms doped rGO with numerous of defective pockets as support coordinating with metal ions has its advantage of high electron transport efficiency. Fischer *et al*. designed nitrogen doped rGO/Ni_7_S_6_ and enabled the practicability of active nickel sites and synergetic catalysis with nitrogen doped rGO in electrochemical water splitting reaction^36^. Other works through heteroatoms doped rGO was devoted to maximizing the electron transmission ability of electrocatalysts with high efficient activity and durability^37–40^. Our approach of improving the OER activity of cobalt sulfides with enhanced the electron transfer and exposed electrocatalytic active sites is designing configuration. Hierarchical structures constructed by ultrathin two-dimensional nanosheets *in-situ* growth on heteroatoms doped rGO is in favour of exposing more active sites, and also shortening the distance of charge diffusion. Therefore, *in-situ* growth of ultrathin nanosheets on N, S doped rGO is a prospective way to promote oxygen evolution performance.

Herein, we integrated a new way of constructing Co_9_S_8_/N,S-rGO hierarchical structures through polyol refluxing, sulfurization and calcination process. This Co_9_S_8_/N,S-rGO hierarchical architectures which Co_9_S_8_ nanosheets with thickness of 3∼4 nm vertically and densely grew on N,S-rGO nanosheets were favoured for exposing surface actives sites and facilitating electron transport, which exhibited high-efficient oxygen evolution electrocatalytic performance and robust durability for electrolytic water in alkaline media.

## Results and Discussion

**Figure 1:**
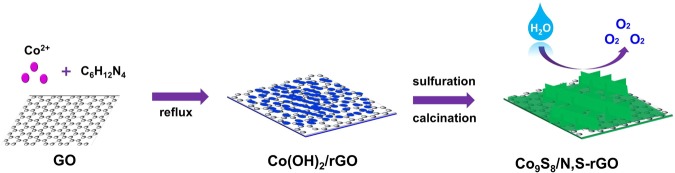
Schematic illustration of synthetic routes for Co_9_S_8_/N,S-rGO hierarchical structures.

Co_9_S_8_/N,S-rGO hierarchical structures were prepared *via* a two-step method as illustrated in Fig. 1. Typically, during the refluxing process, GO was reduced to rGO by ethylene glycol. Oxygenic groups (such as hydroxyl or carboxyl) in the GO can absorb and form bonds with Co^2+^ ^39,40^. The Co^2+^ on the surface of rGO reacted with hexamethylenetetramine under the alkaline condition, forming sandwiching Co(OH)_2_/rGO. The SEM and TEM images of Co(OH)_2_/rGO (Fig. S1), revealed that ultrathin Co(OH)_2_ nanoflakes were uniformly on the surface of two-dimensional rGO, demonstrating the formation of sandwiched structures. XRD patterns, Elemental mappings and Raman spectroscopy indicated that the successful growth of Co(OH)_2_/rGO sandwiched structure (Figs S2–S4). During the sulfuration procedure, TAA slowly released S^2−^ions, converting Co(OH)_2_/rGO to Co_9_S_8_/rGO^41^. To increase the crystallinity of Co_9_S_8_, Co_9_S_8_/rGO was further annealed at 350 °C for 2 h under Ar flow. After the annealing process, N, S atoms *in-situ* doped in rGO and Co_9_S_8_/N,S-rGO hierarchical structure formed.

**Figure 2:**
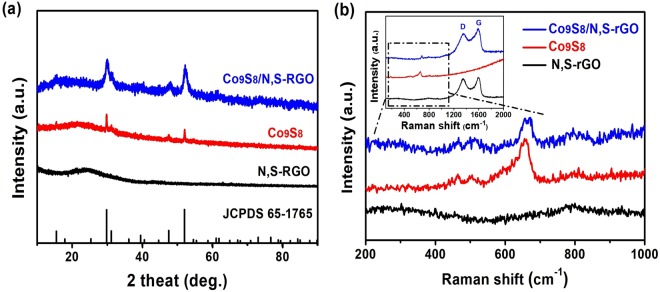
(**a**) XRD patterns and (**b**) Raman spectra of Co_9_S_8_, N,S-rGO and Co_9_S_8_/N,S-rGO.

In Fig. 2a, presented the XRD pattern of Co_9_S_8_/N,S-rGO, in which diffraction peaks were readily indexed to cubic phase Co_9_S_8_ (JCPDS no. 65–1765). To further investigate the role of interaction between Co_9_S_8_ and N,S-rGO, N,S-rGO nanosheets (Fig. S5) and Co_9_S_8_ nanospheres (Fig. S6) were also synthesized via the same synthetic route with the exception that the graphene oxides or Co(Ac)_2_ precursors were not involved in the synthesis, respectively. The broad peak at 24° revealed the representative carbon peaks in N,S-rGO. For Co_9_S_8_ sphere, all the diffraction peaks were the same as Co_9_S_8_/N,S-rGO and corresponded to cubic Co_9_S_8_ (JCPDS no. 65–1765, Fig. 2a). In Fig. 2b, Raman peaks at 459, 503 and 662 cm^−1^ were assigned to Co_9_S_8_ and the observed D bands (1349 cm^−1^), G bands (1591 cm^−1^) were assigned to N,S-rGO, respectively^33,42–46^.

**Figure 3:**
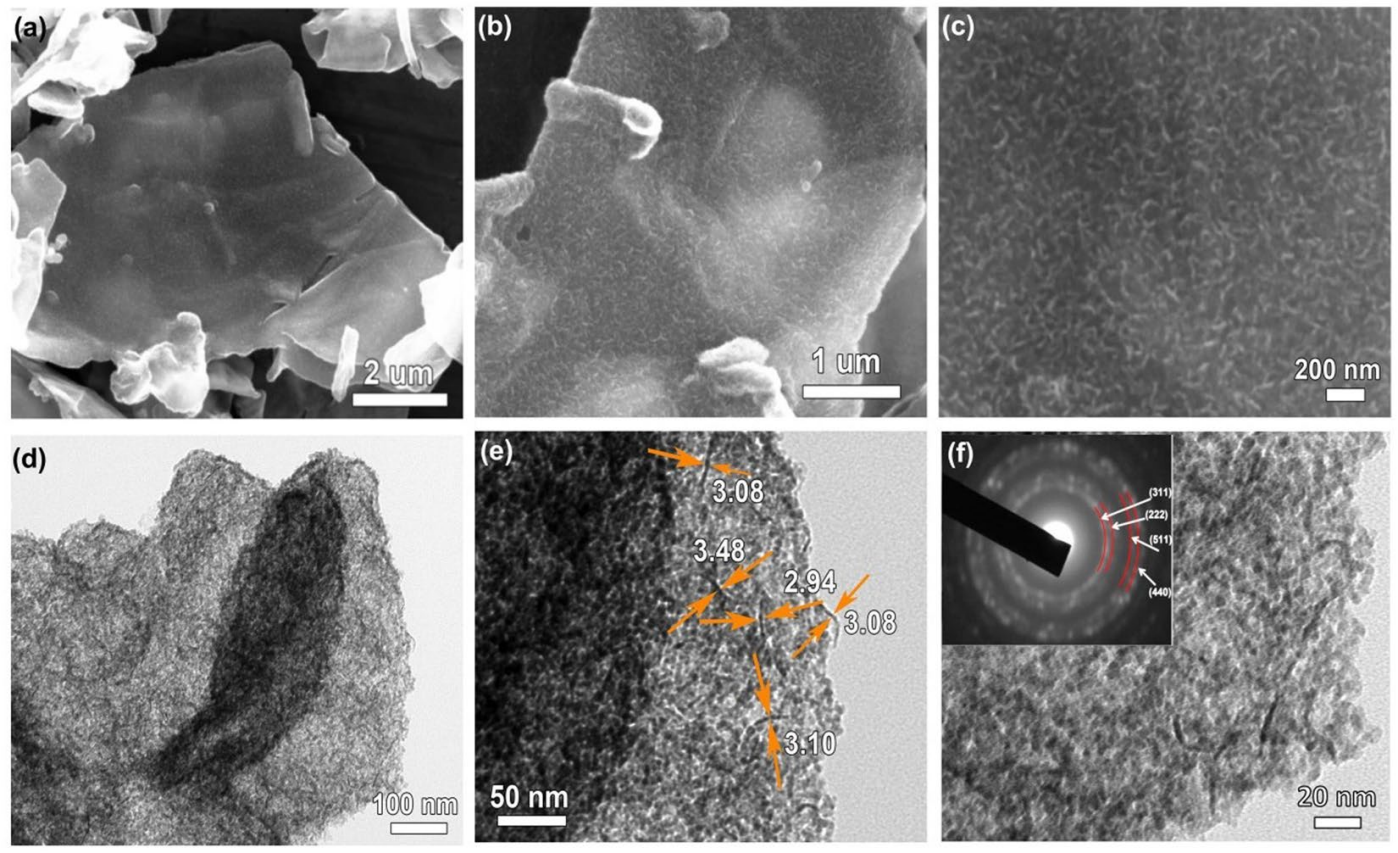
Structural characterization of Co_9_S_8_/N,S-rGO hierarchical structures obtained after sulfuration and calcination process. (**a**–**c**) Low and high magnification SEM images; (**d**) TEM images and (**e**,**f**) high magnification TEM images show the diameters of ultrathin Co_9_S_8_ nanosheets vertically aligned on the surface of N,S-rGO (inset is SAED pattern of cubic phase Co_9_S_8_).

The morphological information of Co_9_S_8_/N,S-rGO was gained from SEM images in Fig. 3a,b, where the sandwiched structure was maintained after sulfuration. In Fig. 3c, the ultrathin Co_9_S_8_ nanosheets aligned vertically and grew densely on the surface of N,S-rGO. TEM images displayed the internal structure of Co_9_S_8_/N,S-rGO in Fig. 3d and e, in which the surface of N,S-rGO was evenly and vertically aligned by ultrathin Co_9_S_8_ nanosheets with diameters of ∼3–4 nm. SAED pattern taken on Co_9_S_8_/N,S-rGO was displayed in Fig. 3f inset, in which all diffraction rings were corresponded to (311), (222), (511) and (440) planes of cubic Co_9_S_8_. These results illustrated a polycrystalline nature of Co_9_S_8_/N,S-rGO, which is in agreement with the XRD results in Fig. 2a. The element mapping revealed the co-existence and uniform dispersion of C, N, O, S and Co (Fig. S7).

**Figure 4:**
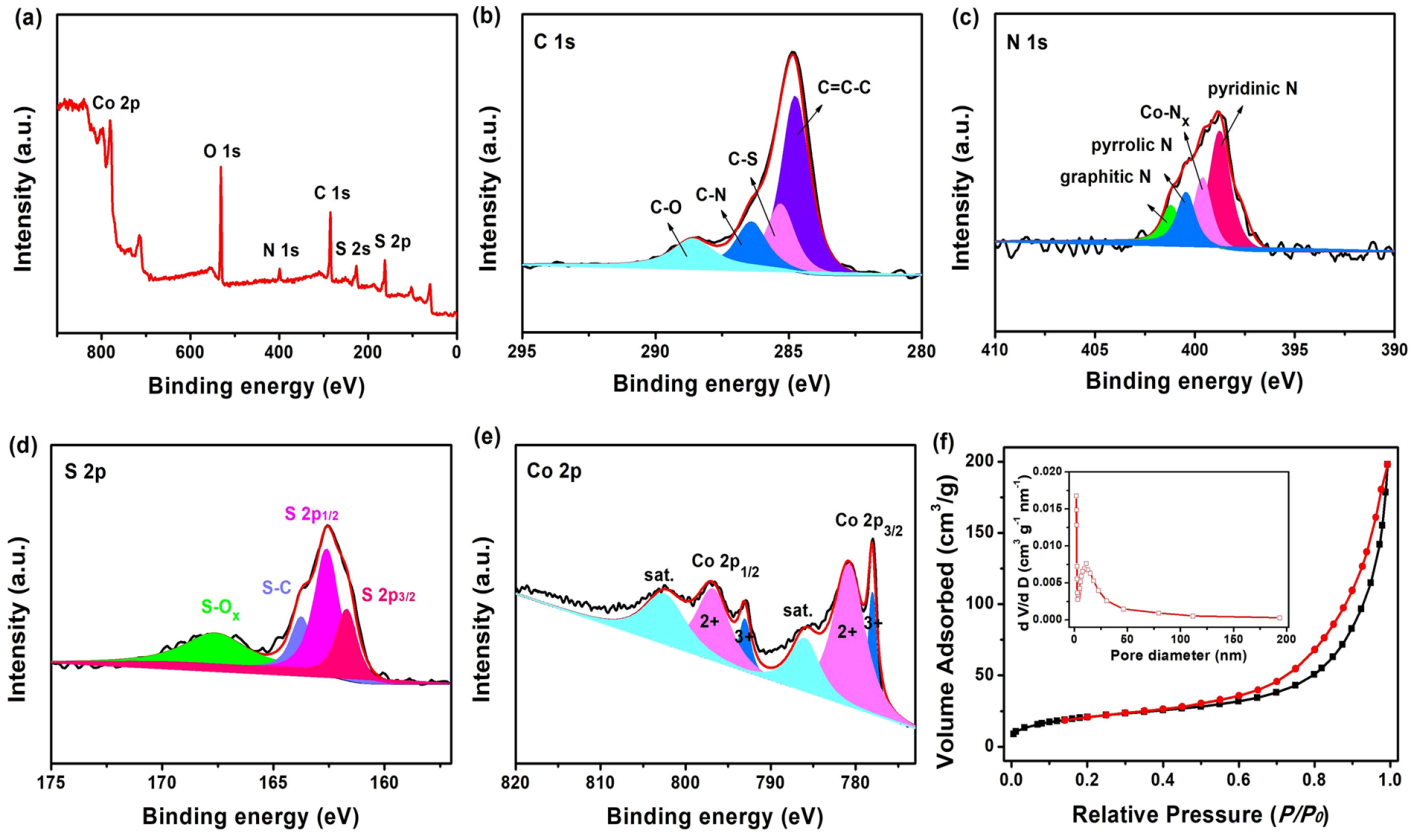
(**a**) High-resolution XPS survey spectra; (**b**) C1s; (**c**) N 1s; (**d**) S 2p; (**e**) Co 2p and (**f**) N_2_-adsorption-desorption isotherm and pore size distribution plots (insert) of Co_9_S_8_/N,S-rGO.

To analyse the chemical states of different elements in Co_9_S_8_/N,S-rGO, X-ray photoelectron spectrometry (XPS) measurement was performed. As shown in the full spectrum in Fig. 4a, C, O, N, S and Co were probed, inferring the co-existence of these elements. In Fig. 4b, the spectra of C 1 s were deconvoluted into four major functional groups, corresponding to the non-oxygenated C-C bond (284.8 eV), C-O bond (288.6 eV), C-S (285.4 eV) and C-N bond (286.4 eV) in rGO^40^. In Fig. 4c, four peaks in N 1 s spectrum were respectively assigned to pyridinic N (398.8 eV), Co-N_*x*_ (399.7 eV), pyrrolic N (400.5 eV) and graphitic N (401.3 eV), indicating that N was successfully doped into rGO^29,47^. The S 2p XPS spectra (Fig. 4d) were attributed to the typical S 2p_3/2_ (161.7 eV) and S 2p_1/2_ (162.6 eV) of cobalt-sulfide bonds. The covalent S-C bond peak was located at 163.8 eV^48^. The peak at 167.7 eV was corresponded to S-O_*x*_, in which contributed to the partial oxidization of sulfur in ambient atmosphere^48^. The XPS survey spectra indicated the successful doping of N and S in rGO and surface atom contents was calculated to be 6.19 and 14.45 at%, respectively. The doublet peaks of Co 2p_3/2_ and 2p_1/2_ with binding energies centered at 778.0, 792.9 eV and 780.7, 796.7 eV were ascribed to Co^3+^ and Co^2+^ state (Fig. 4e)^49,50^. All the above spectra confirmed the formation of Co_9_S_8_/N,S-rGO. The specific surface area of the as-prepared Co_9_S_8_/N,S-rGO was obtained from Brunauer-Emmett-Teller (BET) measurement (Fig. 4f). The Co_9_S_8_/N,S-rGO afforded a high BET specific surface area of 77.2 m^2^·g^−1^. This highly open architecture with ultrathin Co_9_S_8_ nanosheets vertically aligned on N,S-rGO should be helpful for fully accessing electrolyte and exposing catalytic active sites during electrochemical reaction^51,52^. To further investigate the structure advantages in Co_9_S_8_/N,S-rGO, Co_9_S_8_ nps/N,S-rGO were prepared by same steps except the addition of hexamethylenetetramine, as shown in Figs S8, 9. In Co_9_S_8_ nps/N,S-rGO, cubic Co_9_S_8_ nanoparticles were uniformly distributed on the N,S-rGO.

**Figure 5:**
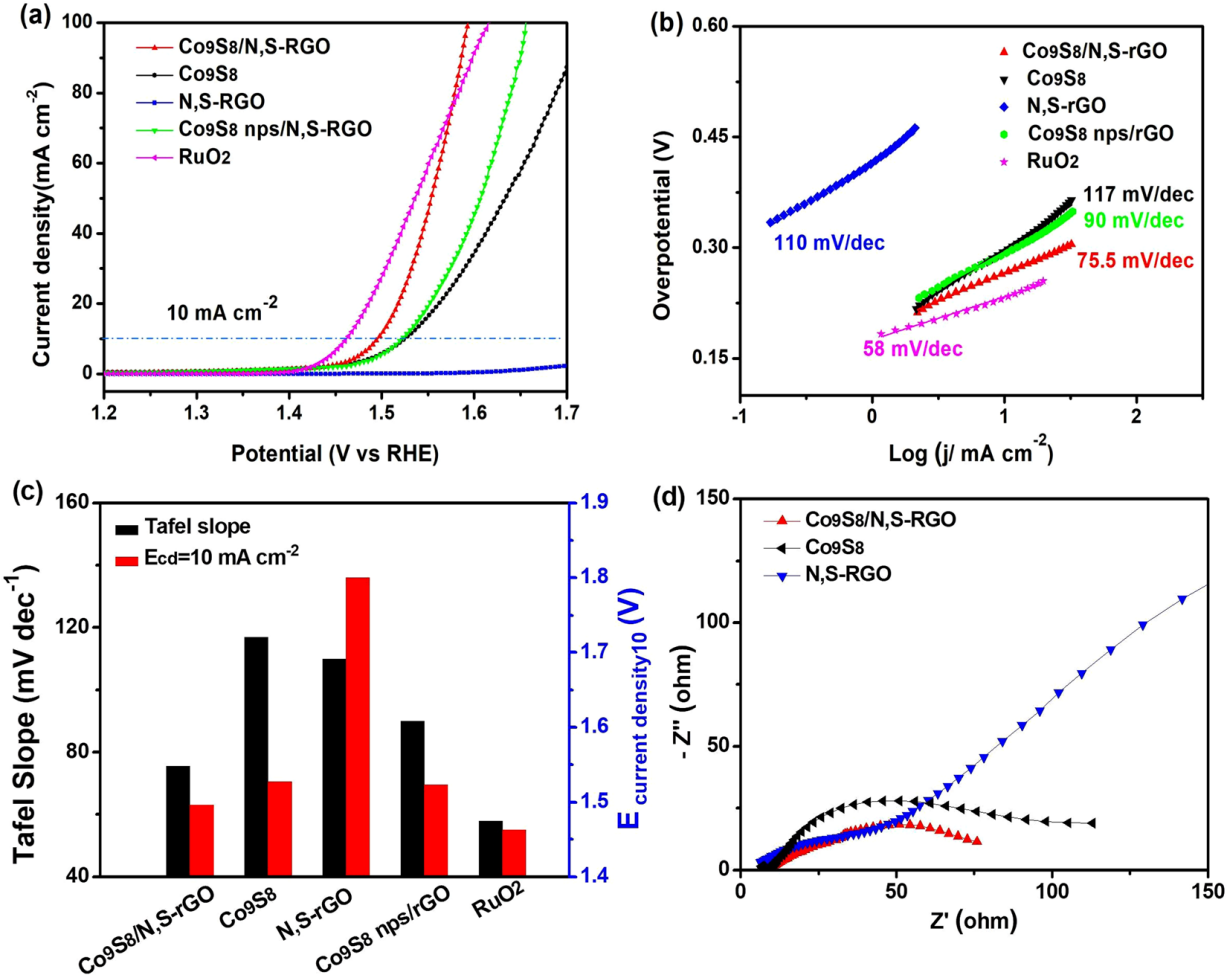
(**a**) IR-corrected polarization curves; (**b**) Corresponding Tafel slopes; (**c**) Summary of Tafel slope and overpotential at current density of 10 mA cm^−2^ for Co_9_S_8_/N,S-rGO, Co_9_S_8_, N,S-rGO, Co_9_S_8_ nps/N,S-rGO and RuO_2_ in 1 M KOH and (**d**) Electrochemical impedance spectra with overpotential of 274.7 mV for Co_9_S_8_/N,S-rGO, Co_9_S_8_, and N,S-rGO.

The OER electrocatalytic activity of Co_9_S_8_/N,S-rGO was performed in alkaline electrolyte (1 M KOH) in a typical three electrode configuration. All data was corrected with iR compensation. In the experiment, RuO_2_ nanopowders were used as benchmark catalyst. OER performance of Co_9_S_8_, N,S-rGO and Co_9_S_8_ nps/N,S-rGO were also performed (Fig. 5). The overpotential to achieve a current density of 10 mA cm^−2^ is an important factor to evaluate the oxygen evolution activity of electrocatalysts. According to the linear sweep voltammograms (LSV) curves displayed in Fig. 5a, the overpotential at current density of 10 mA cm^−2^ for Co_9_S_8_/N,S-rGO was only 266 mV, which is much lower than that of Co_9_S_8_ (297 mV) and Co_9_S_8_ nps/N,S-rGO (292 mV), and closed to that of benchmarking RuO_2_ (232 mV). The bare N,S-rGO possessed an overpotential of more than 462 mV even at 2 mA cm^−2^, suggesting that in Co_9_S_8_/N,S-rGO, Co_9_S_8_ is the main contributor for OER electrocatalytic active sites. Moreover, Co_9_S_8_/N,S-rGO with ultrathin Co_9_S_8_ nanosheets vertical growth on N,S-rGO can provide large exposed catalytic active sites, probably accounting for higher activity in comparsion to Co_9_S_8_ nps/N,S-rGO. Such an overpotential is much lower than other cobalt compounds in alkaline media towards OER, such as hierarchical cobalt sulphide^53^, O-CoS_2_-MoS_2_ heteronanosheet^54^, CoO/N-Graphene^55^, *etc*. Tafel slopes were calculated to analyze the OER kinetics of all electrocatalysts. The high OER activity of Co_9_S_8_/N,S-rGO was confirmed by the small Tafel slope (75.5 mV dec^−1^) in Fig. 5b, which was much lower than those of Co_9_S_8_ nps/N,S-rGO (90 mV dec^−1^), Co_9_S_8_ (117 mV dec^−1^) and N,S-rGO (110 mV dec^−1^). The different Tafel slope values of electrocatalysts signified the different electrochemical oxidation pathways and rate-determining steps. The N,S-rGO with the largest Tafel slope value demonstrated the intrinsically inferior oxygen evolution property. Compared with Co_9_S_8_ and Co_9_S_8_ nps/N,S-rGO, Tafel slope of Co_9_S_8_/N,S-rGO showed distinct acceleration of oxygen evolution kinetics arising from the interaction between Co_9_S_8_ and N,S-rGO. The small Tafel slope values demonstrated that the high OER kinetics and low hydroxyl free radical absorption free energy of Co_9_S_8_/N,S-rGO during oxygen evolution. Figure 5c displayed the comparison of Tafel slope and potential data of current density at 10 mA cm^−2^. The potential of Co_9_S_8_/N,S-rGO was 1.496 V *vs*. RHE, smaller than Co_9_S_8_ nps/N,S-rGO, Co_9_S_8_ and N,S-rGO. To further explore the catalytic activities of Co_9_S_8_/N,S-rGO, the mass activity and specific activity were also calculated (Table 1). At overpotential of 0.27 V, the mass activity and specific activity of Co_9_S_8_/N,S-rGO were 27.03 A g^−1^ and 0.035 mA cm^−2^, respectively. These excellent performance mainly attributed to the hierarchical architecture with ultrathin Co_9_S_8_ nanosheets vertically grown on N,S-rGO which can increase the number of catalytic active sites by exposing large effective surface area and facilitate fast electron transport. The synergetic catalysis of ultrathin Co_9_S_8_ nanosheets and N,S-rGO greatly facilitated the intrinsic catalytic activity of Co_9_S_8_ and boosted the sluggish reaction kinetics of oxygen evolution^56^.

**Table 1:** OER activity of Co_9_S_8_/N,S-rGO, Co_9_S_8_ nps/N,S-rGO and Co_9_S_8_.

electrocatalyst	*η* at *J* = 10 mA cm^−2^	Tafel slope [mV dec^−1^]	*C*_dl_ [mF cm^−2^]	mass activity at *η* = 0.27 V [A g^−1^]	specific activity at *η* = 0.27 V [mA cm^−2^]
Co_9_S_8_/N,S-rGO	266	75.5	90	27.03	0.035
Co_9_S_8_ nps/N,S-rGO	292	90	10.9	15.18	—
Co_9_S_8_	297	117	55	15.50	—

Specific activity is normalized to the BET surface area.

EIS measurements were undertaken in the frequency range from 100 kHz to 0.01 Hz (Fig. 5d). The charge transfer resistance (*R*_ct_) was corresponded to the depressed semicircle in high-frequency region. The *R*_ct_ of Co_9_S_8_/N,S-rGO was much lower than Co_9_S_8_ at overpotential of 274.7 mV. The Nyquist spectrum of N,S-rGO was composed by a semicircle at high frequencies and a linear part at low frequencies, revealing a lower charge transfer resistance and favorable of mass transport. These suggested that Co_9_S_8_/N,S-rGO electrode possess much faster charge transfer process and high electron transmission efficiency during the electrochemical reaction. The significantly decreased charge transfer resistance of Co_9_S_8_/N,S-rGO was attributed to the structures advantages and synergetic effect of ultrathin Co_9_S_8_ nanosheets vertical growth on N,S-rGO, which facilitate the charge and electron transfer process.

For comprehending the significant difference in OER catalytic performance between Co_9_S_8_ and N,S-rGO, electrochemical double layer capacitances (*C*_dl_) was applied to calculate the electrochemical surface area (ECSA) of electrocatalysts (Figs S10, 11). The *C*_dl_ of Co_9_S_8_/N,S-rGO (90 mF cm^−2^) was much larger than that of Co_9_S_8_ (55 mF cm^−2^), Co_9_S_8_ nps/N,S-rGO (10.9 mF cm^−2^) and N,S-rGO, signifying Co_9_S_8_/N,S-rGO with more exposing active sites in electrochemical process. The large ECSA for Co_9_S_8_/N,S-rGO was owed to the advantages of unique hierarchical structure with ultrathin Co_9_S_8_ nanosheets vertical growth on N,S/rGO and large exposed catalytic active sites.

**Figure 6:**
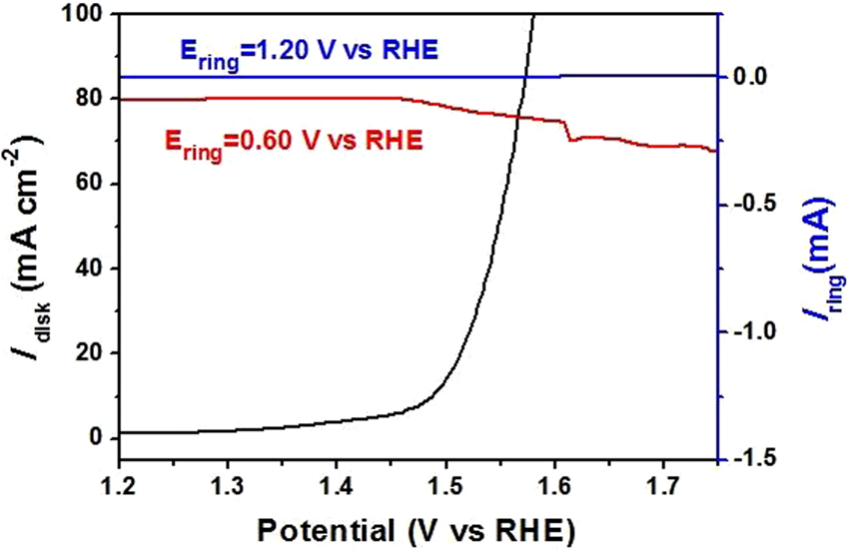
RRDE technique detection of OER products with Pt ring at different potentials for Co_9_S_8_/N,S-rGO in oxygen evolution process.

The rotating ring-disk electrode (RRDE) technique was used to confirm the products formed on electrocatalysts surface during the OER process. As shown in Fig. 6, when the potential of Pt ring electrode is 1.20 V, a microamp-scale oxidation current is recorded (red line), implying negligible production of hydrogen peroxide in the system. Instead, an apparent oxygen reduction reaction (ORR) current was obtained when the potential on Pt ring was at 0.60 V, which verified that the final products were O_2_ during OER process.

**Figure 7:**
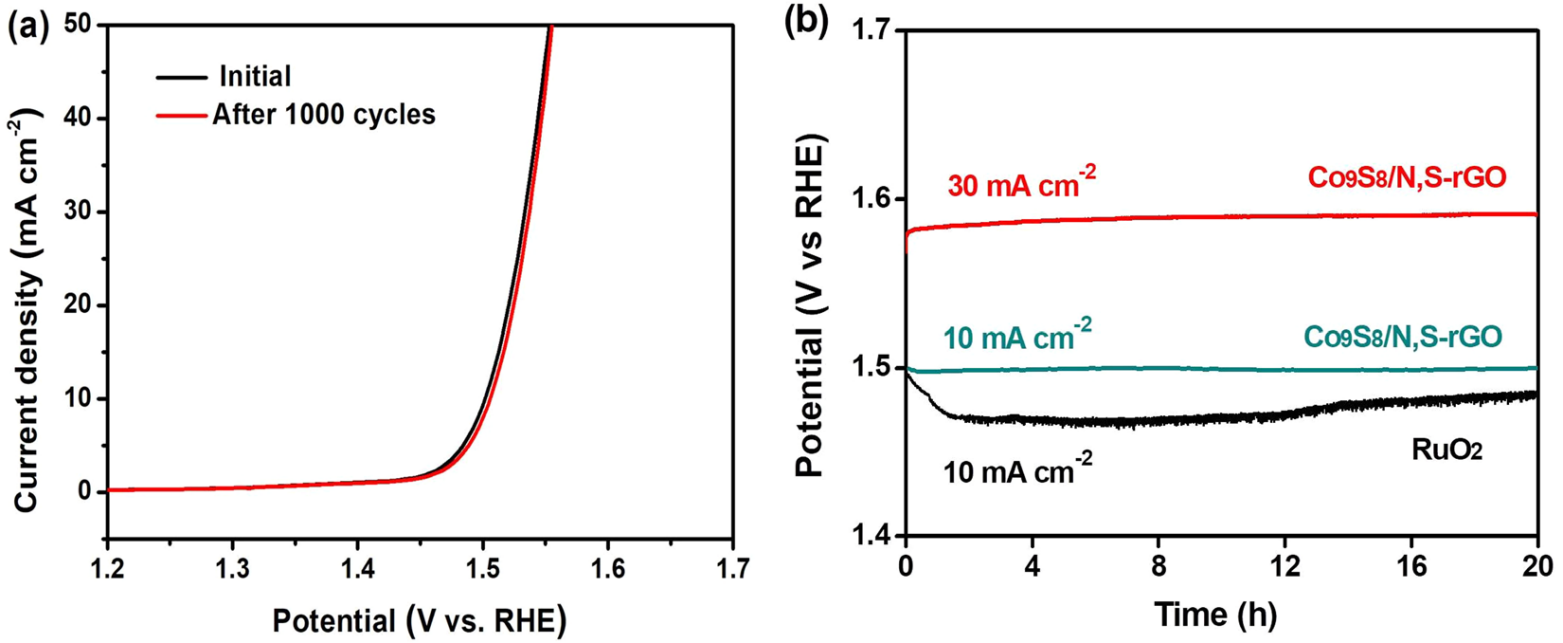
(**a**) Polarization curves of Co_9_S_8_/N,S-rGO before and after 1000 cycles stability test (between 1.0–1.5 V vs. RHE); (**b**) Chronopotentiometry curves of Co_9_S_8_/N,S-rGO and commercial RuO_2_ at a constant current density of 10 and 30 mA cm^−2^.

Electrochemical durability of catalysts is important for practical water splitting applications. Hence, the durability for Co_9_S_8_/N,S-rGO was further evaluated. As shown in Fig. 7a, a continuous potential cycling test (between 1.0–1.5 V vs. RHE) showed a negligible shift in the polarization curves after 1000 cycles, depicting high durability of Co_9_S_8_/N,S-rGO in long term OER under alkaline conditions. The stability of Co_9_S_8_/N,S-rGO was investigated by the chronopotentiometry measurements. As depicted in Fig. 7b, the overpotential at current density of 10 mA cm^−2^ was no obvious decay after running for 20 h, indicating the excellent and stable oxygen evolution ability of Co_9_S_8_/N,S-rGO in long term OER under strong alkaline condition. At higher overpotential of 350 mV, the current density remains stable at ~30 mA cm^−2^ for oxygen evolution over 20 h.

**Figure 8:**
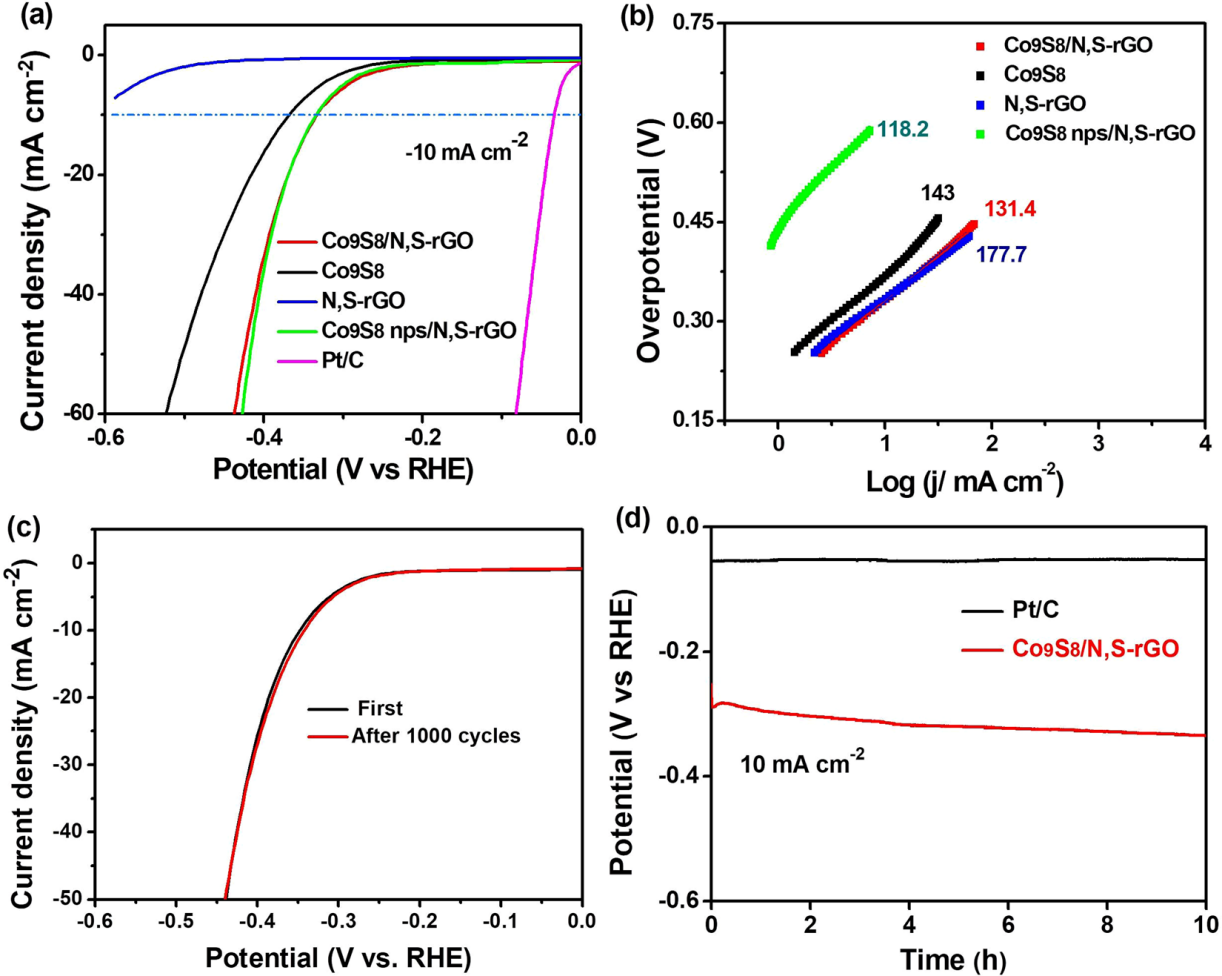
(**a**) Polarization curves; (**b**) Corresponding Tafel slopes of Co_9_S_8_/N,S-rGO, Co_9_S_8_, N,S-rGO and Co_9_S_8_ nps/N,S-rGO for HER in 1 M KOH; (**c**) Polarization curves before and after 1000 cycles stability test of Co_9_S_8_/N,S-rGO; (**d**) Chronopotentiometry curves of Co_9_S_8_/N,S-rGO and commercial Pt/C with constant current density of 10 mA cm^−2^ in 1 M KOH.

Excitingly, besides the high OER electrocatalytic activity, Co_9_S_8_/N,S-rGO also revealed superior catalytic activity towards HER in 1.0 M KOH. As demonstrated in Fig. 8a, a current density of 10 mA cm^−2^ can be achieved at a small overpotential of 332.4 mV, which was much lower than other materials. In comparison with bare N,S-rGO, Co_9_S_8_ (368.2 mV) and Co_9_S_8_ nps/N,S-rGO (334.2 mV), distinctly enhanced activity is observed on Co_9_S_8_/N,S-rGO. The Tafel slope of Co_9_S_8_/N,S-rGO was 131.4 mV dec^−1^, hence the Heyrovsky reaction (H_ads_ + H^+^  + e^−^ → H_2_) is the rate determining step (Fig. 8b)^57^. The N,S-rGO with the highest Tafel slope value demonstrated the intrinsically poor hydrogen evolution property. The Tafel slope value of Co_9_S_8_ nps/N,S-rGO may be restricted by the low exposed electrochemical active surface area. In addition, the almost overlapping LSV curves in Fig. 8c indicated that Co_9_S_8_/N,S-rGO can retain its electrocatalytic activity after 1000 cycles. These results were further verified by the chronoamperometry test that the potential has no obvious increase after 10 h (Fig. 8d), implying the high durability of commercial Pt/C and Co_9_S_8_/N,S-rGO towards HER. By contrast, Co_9_S_8_/N,S-rGO basically retained the initial potential towards HER even after 10 h.

## Conclusions

In summary, Co_9_S_8_/N,S-rGO was fabricated by a facile and cost-effective way as promising substitutes for noble metal OER electrocatalysts. The unique Co_9_S_8_/N,S-rGO hierarchical structure with ultrathin Co_9_S_8_ nanosheets vertically grown on the surface of N and S co-doped reduced graphene oxide has superiority of large exposed electrocatalytic active sites and efficient electron transfer efficiency towards enhancing OER kinetics. This work demonstrates that Co_9_S_8_/N,S-rGO has highly efficient and durable electrocatalytic performance for stable oxygen evolution and moderates hydrogen evolution activity in alkaline condition. This can provide scientific guidance for future research work and understanding the role of electrocatalysts in the electrochemical process.

## Methods

### Materials

Graphene oxide nanosheets (Nanjing XFNANO Materials Tech), Nafion (Sigma-Aldrich), Cobalt acetate, hexamethylenetetramine (C_6_H_12_N_4_), thioacetamide (TAA) and all others reagents were purchased from Sinopharm.

### Materials synthesis

Synthesis of Co(OH)_2_/rGO sandwiched structures: 10 mg of graphene oxide, 1 mmol of Co(Ac)_2_ · 4H_2_O and 1 mmol of hexamethylenetetramine were dispersed in 30 mL ethylene glycol and sonicated for 2 h, respectively. The as-formed supensions were added into a flask and the mixture was stirred at 180 °C for 1 h. After centrifugation and lyophilisation, Co(OH)_2_/rGO sandwiched structures were obtained.

Synthesis of Co_9_S_8_/N,S-rGO hierarchical structures: Co(OH)_2_/rGO sandwiched structures and 1 mmol of thioacetamide were added into 30 mL ethyl alcohol absolute solutions. The mixture was transferred into a Teflon-lined stainless-steel autoclave and heated up at 120 °C for 12 h. The product was washed and centrifugated, followed by carbonization at 350 °C for 2 h under Ar atmosphere.

The fabrications of Co_9_S_8_, N,S-rGO or Co_9_S_8_ nps/N,S-rGO were similar with that of Co_9_S_8_/N,S-rGO but without adding graphene oxide, cobalt acetate or hexamethylenetetramine, respectively.

### Materials characterization

The morphology and crystal structure characterization were performed on a field emission scanning electron microscope (FE-SEM, FEI Quanta 200F), Transmission electron microscopy (TEM, JEOL JEM-2100) and X-Ray Diffraction (XRD, Rigaku D/Max-γB diffractometer). The surface chemical compositions were analysed by X-ray photoelectron spectroscopy (XPS, VG K_*α*_ Probe, Thermo Fisher Scientific). Nitrogen adsorption–desorption measurements were conducted by using a Micromeritics ASAP 2020 system and calculated using BET method.

### Electrochemical measurements

Electrochemical performance of catalysts was evaluated on Wavedrive 20 potentiostat (PINE). A three-electrode configuration was constituted with reference electrode (Ag/AgCl), working electrode (rotating disk electrode (RDE)) and counter electrode (Pt wire). 21 μL of catalyst ink were casted onto glassy carbon (GC) electrode as the working electrode. The overpotentials (*η*), Tafel slope, mass activity (A g^−1^) and specific activity (mA m^−2^) were calculated on the basis of the following formula:

1 $$\eta ={E}_{{\rm{Ag}}/{\rm{AgC}}l}+0.196{\rm{V}}+0.059{\rm{pH}}-1.23{\rm{V}}$$

2 $$\eta =b\,\mathrm{log}\,j+a$$

3 $${\rm{Mass}}\,{\rm{activity}}=J/m$$

4 $${\rm{Specific}}\,{\rm{activity}}=J/(10{S}_{{\rm{BET}}}m)$$

where *j* is the current density (mA cm^−2^); *b* is the Tafel slope (mV dec^−1^); *J* (mA cm^−2^) denotes the current density at *η* = 0.27 V; *m* denotes the catalyst loading (0.37 mg cm^−2^); *S*_BET_ (m^2^ g^−1^) is the BET surface area. Electrochemical impedance spectroscopy (EIS) measurements were carried out in the frequency range of 0.01 Hz to 100 kHz with constant potential of 1.5 V *vs*. RHE.

## Electronic supplementary material

Supporting information
