# Supplementary material for: Ultrathin Co9S8 nanosheets vertically aligned on N,S/rGO for low voltage electrolytic water in alkaline media
Source: Sci Rep. 2019 Feb 13;9:1951. doi: 10.1038/s41598-018-35831-4 (PMC6374427; doi:10.1038/s41598-018-35831-4)
Supplement: Supplementary file 1 — Supporting information [file 41598_2018_35831_MOESM1_ESM.docx]

*Supplementary materials for*

**Ultrathin Co_9_S_8_ nanosheets vertically aligned on N, S/rGO for low voltage electrolytic water in alkaline media**

Huan Liu*^a,b^*, Cheng-Yan Xu*^a,b*^*, Yue Du*^c^*, Fei-Xiang Ma*^a,b^*, Yue Li*^a,b^*, Jing Yu*^a,b^*, Liang Zhen*^a,b,c^*^*^

*^a^* MIIT Key Laboratory of Advanced Structural-Functional Integration Materials & Green Manufacturing Technology, School of Materials Science and Engineering, Harbin Institute of Technology, Harbin 150001, China

*^b^* MOE Key Laboratory of Micro-Systems and Micro-Structures Manufacturing, Harbin Institute of Technology, Harbin 150080, China

*^c^* School of Materials Science and Engineering, Harbin Institute of Technology (Shenzhen), Shenzhen 518055, China

* Corresponding authors. E-mail: cy_xu@hit.edu.cn; lzhen@hit.edu.cn


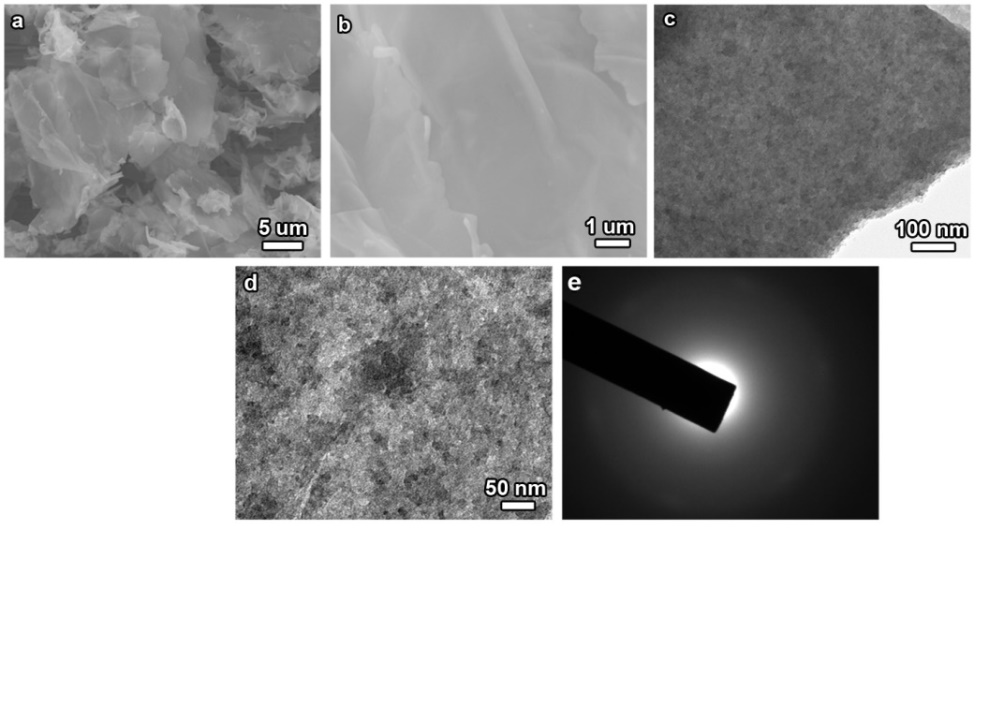


**Figure S1.** Structural characterization of Co(OH)_2_/rGO sandwich structures obtained by ethylene glycol refluxing process (a, b) SEM images; (c, d) TEM images and (e) corresponding SAED pattern.


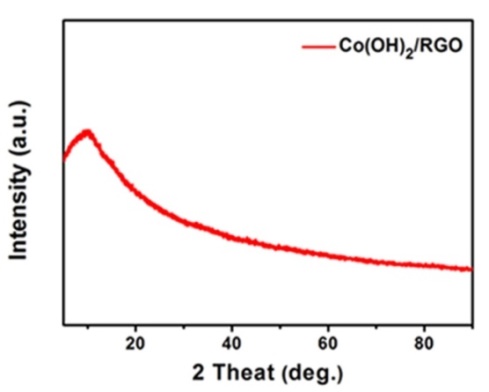


**Figure S2.** XRD pattern of Co(OH)_2_/rGO sandwich structures.


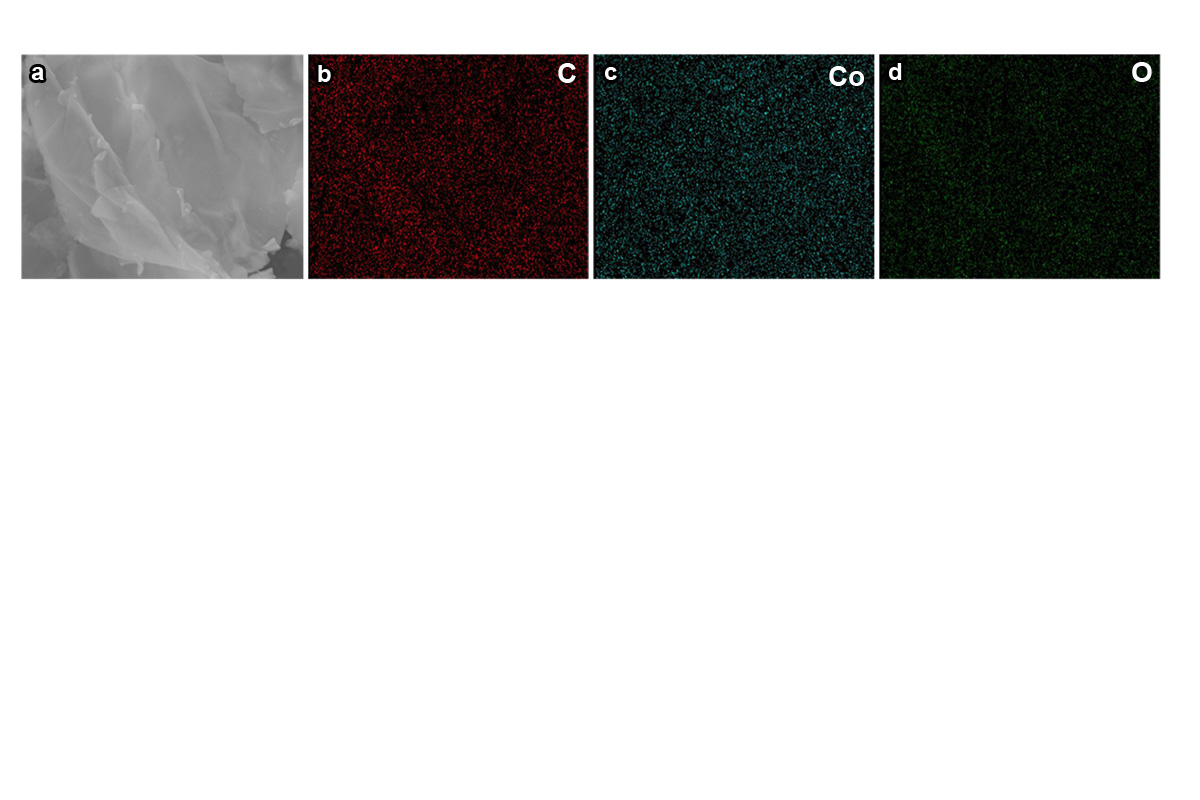


**Figure S3.** EDS mapping of Co(OH)_2_/rGO sandwich structures.

**Figure S4.** Raman spectras of Co(OH)_2_/rGO and Co_9_S_8_/rGO hierarchical structures.

In Figure S4, the Raman spectras of Co(OH)_2_/rGO and Co_9_S_8_/rGO hierarchical structures were exhibited. The D, G bands of graphene were observed which indicated that all the rGO were existed in and hierarchical structures were successfully synthesized. The peaks approximately at 670 cm^-1^ correspond to the Co_9_S_8_.


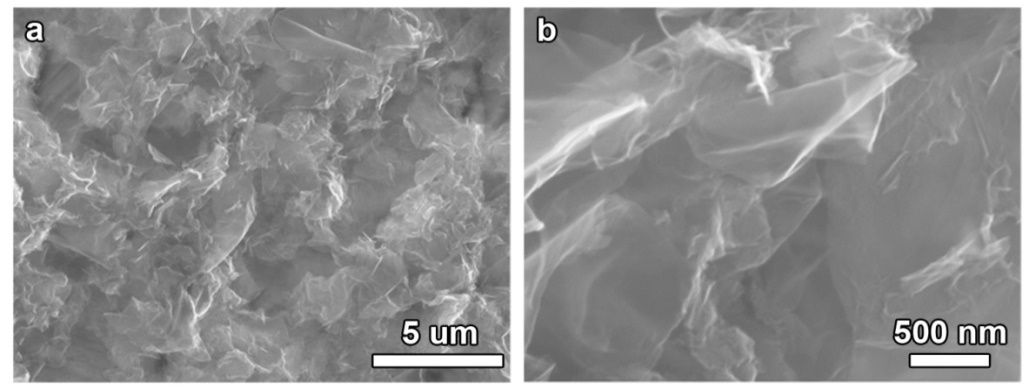


**Figure S5.** SEM images of N,S-rGO structures at different magnifications.

N,S-rGO structures were prepared by the same steps with Co_9_S_8_/N,S-rGO hierarchical but without adding Co(Ac)_2_. N,S-rGO also kept the original morphology of graphene.


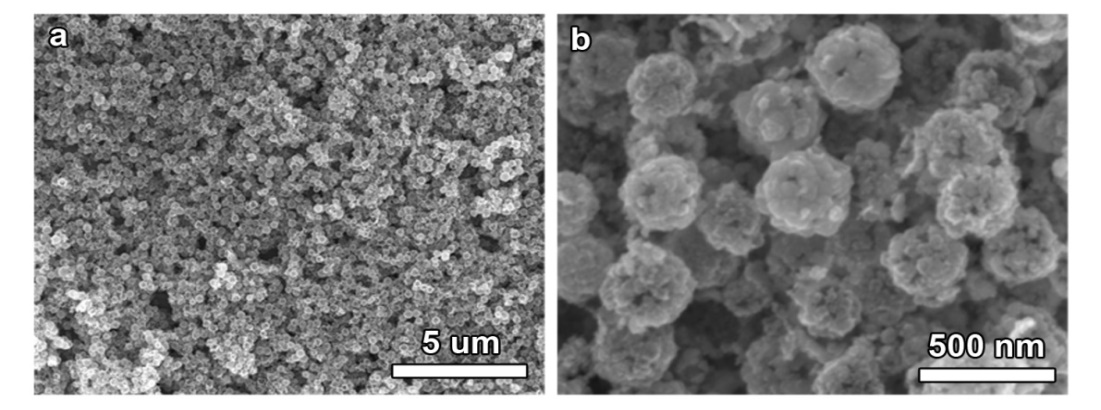


**Figure S6.** SEM images of Co_9_S_8_ nanosphere structures at different magnifications.

Co_9_S_8_ nanosphere structures were prepared by the same steps with Co_9_S_8_/N,S-rGO hierarchical but without adding of graphene oxides.


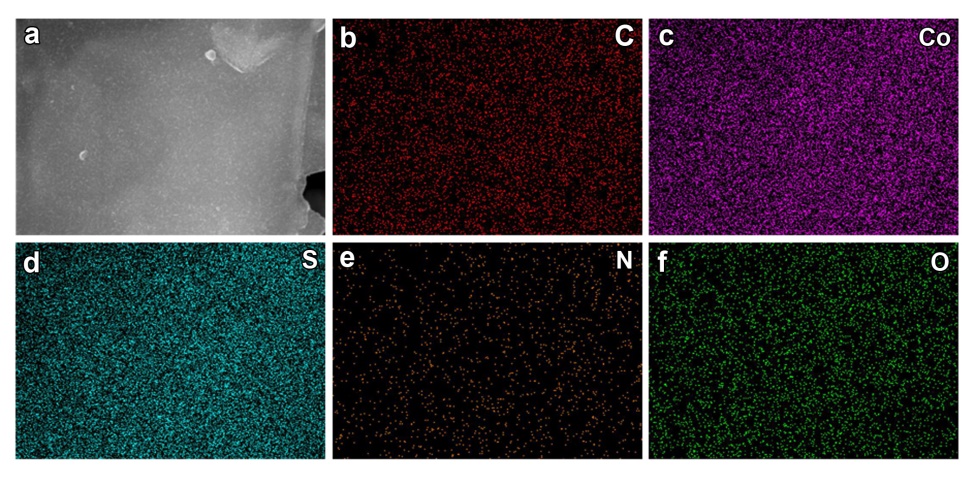


**Figure S7.** EDS mapping of Co_9_S_8_/N,S-rGO hierarchical structures.

**Figure S8.** (a) XRD pattern and (b) Raman spectra of Co_9_S_8_ nps/N,S-rGO hierarchical structures.

Co_9_S_8_ nps/N,S-rGO hierarchical structures were prepared by the same steps with Co_9_S_8_/N,S-rGO hierarchical but without adding hexamethylenetetramine. In Figure S8, all the diffraction peaks observed were cubic Co_9_S_8_ (JCPDS no. 65-6801) in the Co_9_S_8_ nps/N,S-rGO hierarchical structures.


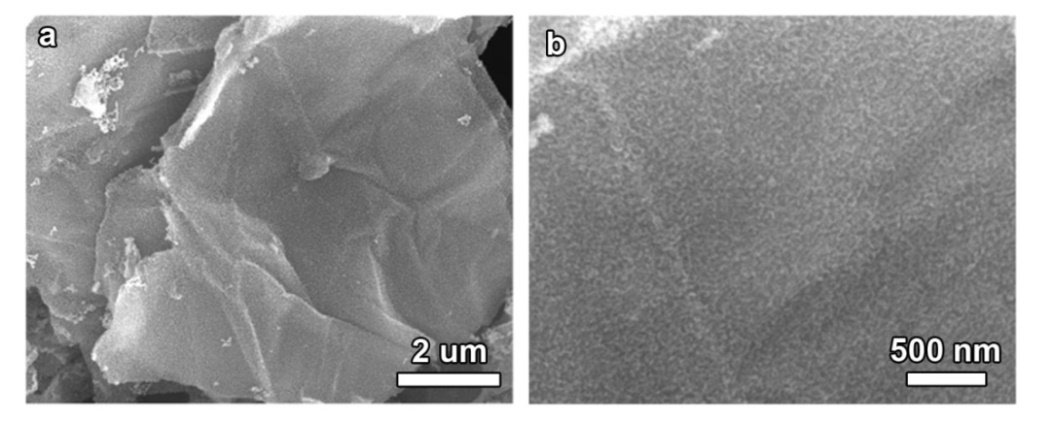


**Figure S9.** SEM images of Co_9_S_8_ nps/N,S-rGO hierarchical structures at different magnifications.

**Figure S10.** Charging current density difference (Δ*j* = *j*_a_ - *j*_c_) plotted against scan rate for Co_9_S_8_/N,S-rGO; Co_9_S_8_; N,S-rGO and Co_9_S_8_ nps/N,S-rGO electrocatalysts.

The effective surface areas of catalysts were compared by estimating their electrochemical double layer capacitances (*C*_dl_) with CV scan. CV curves were performed at a potential range of 1.2−1.3 V *vs.* RHE where no obvious electrochemical features corresponding to the Faradaic current were observed. The capacitive currents at 1.26 V *vs.* RHE were plotted against the scan rate.

**Figure S11.** CV curves of (a) Co_9_S_8_/N,S-rGO; (b) Co_9_S_8_; (c) N,S-rGO and (d) Co_9_S_8_ nps/N,S-rGO in 1M KOH with different scan rates (5, 10, 15, 20, 30 mV s^-1^).
